# Altitude-dependent climate impacts and economic feasibility of alternative fuels in aviation from 2025 to 2050

**DOI:** 10.1016/j.isci.2025.113323

**Published:** 2025-08-07

**Authors:** Qiang Cui, Xu-jie Sun, Xing-yu Tang, Ying Zhou, Yu-xin Zhang, Ye Li

**Affiliations:** 1 School of Economics and Management, Southeast University, Nanjing 211189, China; 2 School of Business Administration, Nanjing University of Finance and Economics, Nanjing, China

**Keywords:** environmental science, applied sciences, economics

## Abstract

The aviation industry plays an increasing role in climate change due to emissions at cruise altitudes. This study combines projections from the Aviation Integrated Model (AIM) and simulations from the Aviation-FAIR model to assess greenhouse gas concentrations, radiative forcing, and temperature effects across emission altitudes from 500 to 40,500 ft during 2025–2050. The results reveal that climate impact intensifies with altitude and peaks at 34,500 ft. In addition, a cost-benefit analysis evaluates the use of sustainable aviation fuel (SAF) and hydrogen energy in civil aviation. By 2050, offsetting the costs of these fuels will require $354.44 billion for SAF and $1,888.44 billion for hydrogen. This study assesses the altitude-dependent climate effects of aircraft emissions and the economic feasibility of alternative aviation energy technologies, highlighting both their mitigation potential and cost-related limitations.

## Highlights

• This study applies the Aviation-FAIR model to simulate climate effects
• A nonlinear relationship between emission altitude and greenhouse gas concentration
• A cost-benefit analysis of adopting SAF and hydrogen energy is done
• By 2050, SAF and hydrogen will require $354.44 billion and $1,888.44 billion, respectively, in revenue to cover costs

## Graphical abstract

**Figure:**
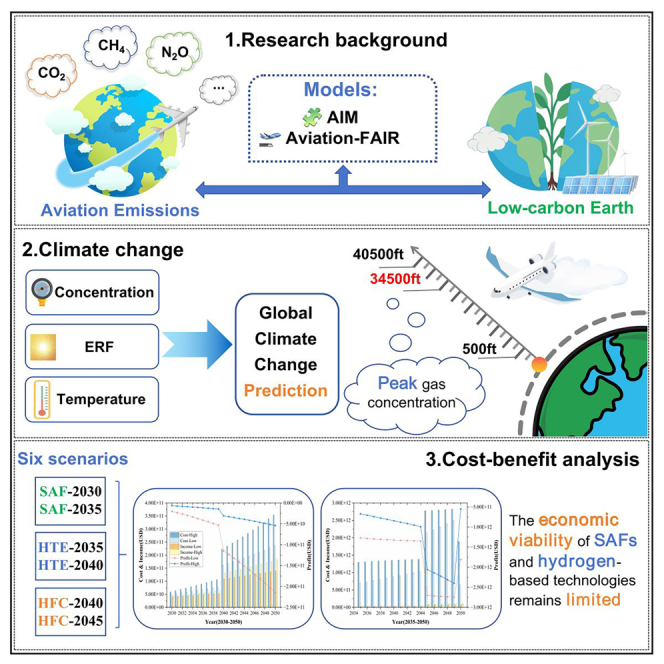

## Introduction

Since the turn of the 21st century, the aviation industry has experienced remarkable growth, reaching approximately 4.5 billion passengers in 2019.^1^ However, the COVID-19 pandemic led to a sharp decline in aviation operations in 2020, with numerous flights canceled in most countries.^2^ As anti-epidemic policies were gradually refined, and borders reopened, the aviation industry has steadily recovered. By 2022, the total number of passengers carried on scheduled flights had risen to 3.3 billion, and the International Air Transport Association forecasted that the global passenger traffic would reach 4 billion by 2024,^3^ surpassing pre-pandemic levels. In line with this prediction, the industry’s total revenue passenger kilometer (RPK) increased by 10.4% year-on-year (YoY) in 2024, exceeding the 2019 levels by 3.8%. Available seat kilometers (ASK) also showed an upward trend, growing by 8.7% YoY. International passenger traffic reached a record high, growing by 13.6% YoY in 2024 and surpassing the 2019 levels by 0.5%, despite ongoing conflicts in some parts of the world affecting airspace. Meanwhile, according to data published by the International Civil Aviation Organization (ICAO), from January to November 2024, RPK stood at 7,589 billion passenger kilometers and ASK totaled 6,911 billion seat-kilometers.^4^ Aviation accounts for approximately 2.5% of the global carbon emissions and 12% of carbon emissions from all transport sources. Before the pandemic, the ICAO had predicted that carbon emissions from international aviation could double by 2050 compared with the 2015 levels.^1^ In addition to carbon dioxide, emissions from aircraft, including nitrogen oxides, water vapor, sulfates, and soot particles at high altitudes, can significantly impact the climate.

Aviation is one of the most energy-intensive forms of consumption,^5^ with aircrafts being among the most carbon-intensive modes of travel.^6^ According to a previous study, the aviation industry historically contributed approximately 4% to the observed anthropogenic climate warming.^7^^,^^8^ The aviation industry’s impact on climate change is primarily attributed to a wide variety of emissions. The release of CO_2_, methane (CH_4_), nitrogen oxides (NOx), water vapor, soot, sulfate aerosols, and contrails disrupts the radiative budget of the Earth’s atmosphere,^9^^,^^10^ leading to changes in the climate system. Among these pollutants, CO_2_ is the largest contributor to climate change, as it further leads to the formation of a “greenhouse effect enhancement layer,” enhancing the ability to retain the heat of Earth and ultimately resulting in global warming.^11^^,^^12^ The influence of non-CO_2_ emissions should not be neglected, with approximately two-thirds of aviation’s radiative forcing attributed to non-CO_2_ effects.^13^ Aircraft-produced contrail cirrus clouds contribute to anthropogenic climate change.^14^ Contrails and the resulting increased cloud cover forming contrail cirrus produce a positive net (warming) effective radiative forcing (ERF) term, consistent with the climate effects of carbon dioxide and nitrogen oxide emissions.^15^^,^^16^ Therefore, aviation emissions, including carbon dioxide and non-CO_2_ emissions such as nitrogen oxides, as well as cloud effects, remain key focuses in research on anthropogenic climate change and policy discussions.^17^ In addition to these effects, short-lived climate forcers also increase greenhouse gas (GHG) concentrations, further driving global temperature rises.^18^^,^^19^ As a result, aviation’s impact on climate change will continue to intensify unless appropriate mitigation measures are taken. Assuming the industry returns to pre-pandemic levels, aviation emissions are projected to contribute approximately 0.1°C to global temperature rise by 2050.^20^^,^^21^ The impact of global aviation emissions on climate change cannot be longer neglected, making the exploration of emission reduction pathways a critical issue in addressing global temperature rise and promoting the sustainable development of the aviation industry. Existing research primarily focuses on optimization of aircraft fuel and carbon trading mechanisms. The European Union Emissions Trading System (EU ETS), initiated in 2005, has only had limited effectiveness in controlling aviation emissions, as it mainly played a role in regulating domestic aviation emissions without alleviating international aviation pollution.^22^ From the perspective of energy transition, applying synthetic fuels derived from biomass, synthetic fuels from green hydrogen and atmospheric CO_2_, or directly using green liquid hydrogen in aviation presents great potential for emission reduction.^23^^,^^24^ Among these novel aviation fuels, sustainable aviation fuel (SAF) stands out as a particularly effective solution for a greater capability of emission reduction rather than relying solely on the EU ETS. SAF is considered as one of the best strategies to achieve net-zero emissions (NZEs) in 2050.^25^ SAF can be produced through various pathways, with waste biomass feedstocks making a significant contribution to its production for the aviation industry.^26^ In addition to the aforementioned options, reducing the scale of air transportation is also an effective short- to medium-term strategy for reducing emissions.^27^

The application of alternative energy in the civil aviation industry has become a core issue in the global fight against climate change and the goal of achieving carbon neutrality in the aviation industry. In terms of the application and cost-benefit analysis of alternative energy technologies (such as biofuels, hydrogen energy, electric aviation, etc.) in the civil aviation industry, it is generally confirmed that alternative energy can significantly reduce the life cycle carbon emissions of the aviation industry,^23^^,^^28^ but the emission reduction contribution of different technology paths is significantly different.^29^ Improvements in fuel efficiency driven by market forces could address about a quarter of the projected global life cycle CO_2_ equivalent emissions by 2050 and the global aviation industry will need to invest about $1.7 trillion in the energy transition over the next 30 years, because of which aircraft operating costs could increase by 10%–16%.^25^ Alternative fuel costs are two to five times the price of conventional jet fuel. This price gap is mainly due to the high investment in the production process, and equipment maintenance costs (such as regular maintenance of reactors, separators, and purification systems) continue to increase operating cost pressure.^30^^,^^31^ In terms of the risks, economics, and environmental benefits of electrification of SAF production, SAF produced using CO_2_ is expected to reduce carbon intensity by 50%–94% compared with fossil fuels. To further reduce carbon intensity, the key is to use high-purity CO_2_ raw materials and low-energy conversion paths and combine renewable energy to handle the CO_2_ capture and purification process.^32^ Under the optimistic hydrogen cost assumption, the operating costs of short- and medium-range hydrogen aircrafts are like the kerosene-powered baseline. However, if the price of hydrogen is in the pessimistic range, its operating costs will double. This cost uncertainty directly affects the industry’s willingness to invest.^33^

In aviation studies, the Aviation Integrated Model (AIM) is widely used to analyze flight technology, policy interventions, and other key areas. AIM is a global aviation systems model that simulates interactions among passengers, airlines, airports, and other stakeholders to forecast the impacts of policy decisions and system changes on aviation’s externalities and economic outcomes.^34^ The model comprises seven interconnected modules: Aircraft Technology & Cost, Aircraft Movement, Airport Activity, Air Transport Demand, Global Climate, Air Quality & Noise, and Regional Economies, each focusing on a specific aspect of the global aviation system.^35^ Using data from the 2015 global aviation industry, AIM projects industry trends through 2050 and defines four key scenarios^36^: Ref_Mid, which assumes no major aviation policies or alternative fuel adoption; Pol_Mid, which accounts for the effects of policies such as ICAO’s Carbon Offsetting and Reduction Scheme for International Aviation (CORSIA) (implemented in early 2023), the EU and UK ETS, and moderate demand growth; Pol_Low, which maintains the same policies as Pol_Mid but with a lower demand growth rate; and H2_Mid, which explores hydrogen energy adoption under medium demand growth and fluctuating oil prices.

This article focuses on two key questions. First, in the context of the rapid development of the global aviation industry, to what extent does civil aviation contribute to global climate change? Second, with alternative energy sources being regarded as a crucial pathway for reducing emissions in the sector, what are the potential future costs and benefits associated with their adoption?

Building on the prediction results of the AIM, this study explores the following key areas. First, the Aviation-FAIR (Finite Amplitude Impulse Response) model is employed to estimate the atmospheric concentrations, ERF, and temperature changes at various emission altitudes, ranging from 500 to 40,500 ft, over the period from 2025 to 2050. Second, this study evaluates the costs and benefits of emerging aviation technologies such as SAF, hydrogen turbines, and hydrogen batteries from 2025 to 2050.

## Results

### Changes in atmospheric concentrations across four scenarios

**Figure 1:**
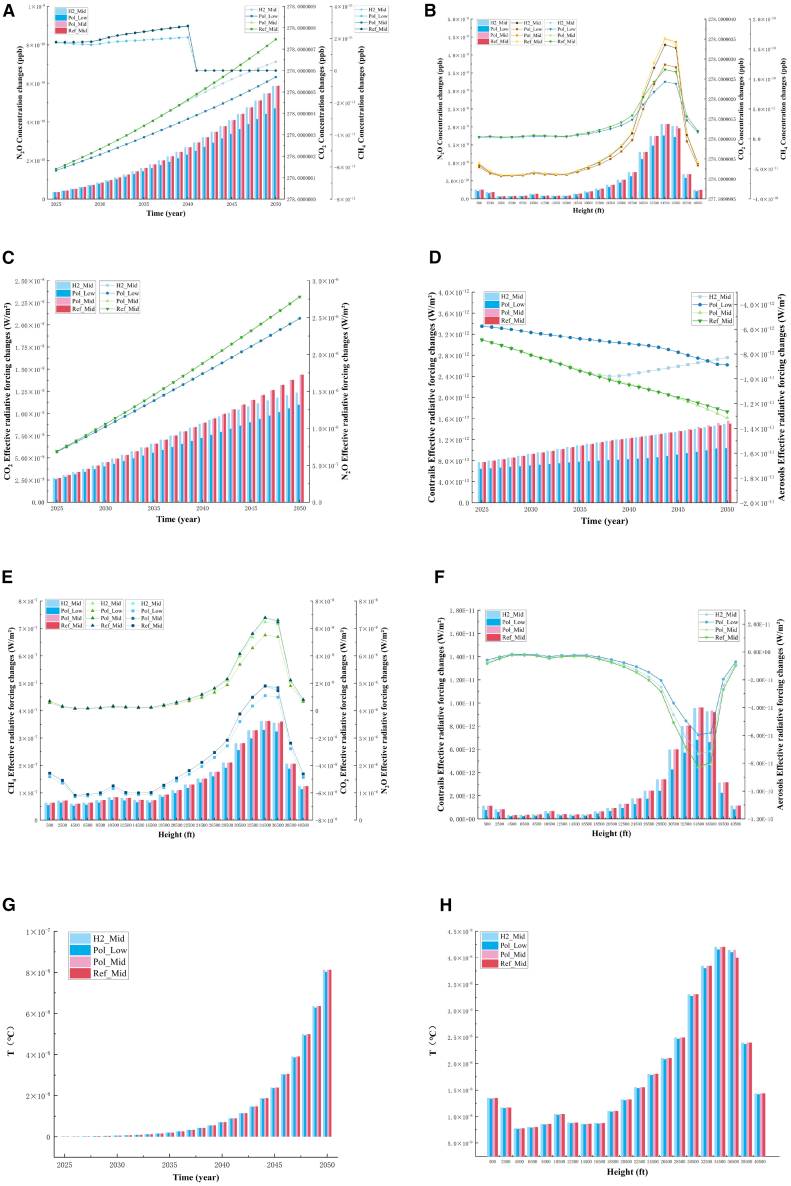
Climate change scenarios (A) Concentration changes of N_2_O, CO_2_, and CH_4_ under four scenarios from 2025 to 2050. (B) Concentration changes of N_2_O, CO_2_, and CH_4_ under four scenarios from 500 to 40,500 ft. (C) ERF changes of CO_2_ and N_2_O under four scenarios from 2025 to 2050. (D) ERF changes of contrails and aerosol under four scenarios from 2025 to 2050. (E) ERF changes of CH_4_, CO_2_, and N_2_O under four scenarios from 500 to 40,500 ft. (F) ERF changes of contrails and aerosol under four scenarios from 500 to 40,500 ft. (G) Temperature changes under four scenarios from 2025 to 2050. (H) Temperature changes under four scenarios from 500 to 40,500 ft.

This study compares the projected changes in CO_2_, CH_4_, and N_2_O concentrations from 2025 to 2050 at emission altitudes ranging from 500 to 40,500 ft. Figure 1A illustrates these trends, showing that CO_2_ and N_2_O concentrations continue to rise across all four scenarios, whereas CH_4_ exhibits a more gradual increase before declining to zero by 2041. The variations in CO_2_ concentrations among the three policy-driven scenarios reveal that Pol_Mid has a limited impact on reducing emissions compared with Ref_Mid, which is not reflected in the figure. The effectiveness of the remaining three scenarios, ranked from strongest to weakest in controlling CO_2_ levels, is Pol_Low, H2_Mid, and Ref_Mid. Both Pol_Low and H2_Mid demonstrate a reduction in CO_2_ concentrations relative to Ref_Mid, suggesting that aviation policies, demand management, and the adoption of hydrogen energy play a crucial role in mitigating CO_2_ emissions. Conversely, the weak control of CO_2_ under the Ref_Mid scenario indicates that, without significant policy interventions or alternative fuel adoption, aviation-related GHG emissions will continue to rise.

Among the scenarios, Pol_Low is the most effective at controlling N_2_O concentrations, followed by Pol_Mid, H2_Mid, and Ref_Mid. By 2050, N_2_O concentrations range between a minimum of 4.70E-10 ppb and a maximum of 5.87E-10 ppb, representing an approximate 20% reduction. CH_4_ concentrations exhibit an increasing trend across all scenarios, with Pol_Low being the only scenario that significantly mitigates CH_4_ emissions compared with Ref_Mid. The differences between Pol_Mid and H2_Mid are relatively minor.

Figure 1B illustrates how CO_2_, CH_4_, and N_2_O concentrations vary with emission altitude, showing a nonlinear trend that peaks at 34,500 ft before declining. At lower altitudes (500–20,500 ft), concentration variations between scenarios are minimal, with CO_2_ levels at approximately 278.00000015 ppb, CH_4_ at 5.35E-11 ppb, and N_2_O at 5.26E-12 ppb. As the altitude increases, concentration differences become more pronounced, peaking at 34,500 ft where CO_2_ reaches ∼278.000003 ppb, CH_4_ reaches ∼1.16E-10 ppb, and N_2_O reaches ∼2.07E-9 ppb. At this altitude, scenario-driven differences are most significant, with Pol_Low demonstrating the most effective control over all three gases, particularly CH_4_.

### Changes in ERF across the scenarios

Figures 1C–1F illustrate the changes in ERF for CO_2_, N_2_O, CH_4_, contrails, and aerosols at emission altitudes ranging from 500 to 40,500 ft between 2025 and 2050 under the four aviation scenarios (see Figures 1 and S2). As shown in Figure 1A, the ERF trends for CO_2_, CH_4_, and N_2_O closely follow their respective concentration trends. In the Ref_Mid scenario, the ERF of these gases exhibits a continuous increase, indicating that without policy interventions or alternative fuels, aviation emissions will have a growing impact on climate change.

Figure 1C depicts the ERF changes for CO_2_ and N_2_O. The CO_2_ ERF follows a linear upward trend, with the H2_Mid and Pol_Mid scenarios showing little difference from Ref_Mid. In Ref_Mid, CO_2_ ERF reaches 2.78E-6 W/m^2^ by 2050, highlighting the need for emission control measures. However, in the Pol_Low scenario, which incorporates aviation policies and reduced demand growth, CO_2_ ERF is contained at 2.49E-6 W/m^2^, representing a 10% reduction compared with Ref_Mid. The N_2_O ERF also follows a linear growth pattern, with control effectiveness ranked from strongest to weakest as Pol_Low, H2_Mid, Pol_Mid, and Ref_Mid. In Ref_Mid, N_2_O ERF rises continuously, reaching 1.43E-8 W/m^2^ in 2050. In H2_Mid, due to the adoption of hydrogen energy, N_2_O ERF is reduced by 14% to 1.23E-8 W/m^2^, whereas the Pol_Low scenario achieves the most significant reduction to 1.10E-8 W/m^2^, a 23% decrease from Ref_Mid, underscoring the importance of aviation policies in mitigating climate impact. As shown in Figure S2, the ERF of CH_4_ exhibits a slow increase from 2025 to 2029, followed by a sharp decline, reaching 0 W/m^2^ after 2041. Although the H2_Mid and Pol_Mid scenarios provide some control over CH_4_ emissions, their impact remains relatively minor compared with Ref_Mid. The Pol_Low scenario, however, achieves the most significant reduction, with CH_4_ ERF reaching 1.96E-7 W/m^2^ in 2040, which is 14% lower than in Ref_Mid (2.27E-7 W/m^2^).

Figure 1D illustrates ERF changes for contrails and aerosols across the four scenarios. The ranking of scenarios in terms of control effectiveness is Pol_Low, H2_Mid, Ref_Mid, and Pol_Mid. Notably, Pol_Mid is less effective in controlling contrail ERF than in mitigating CO_2_ and CH_4_ ERF. Aerosol ERF remains consistently negative, with H2_Mid and Pol_Low demonstrating the strongest control. After 2037, the H2_Mid scenario surpasses Pol_Low in reducing contrail ERF, whereas Pol_Mid achieves lower contrail ERF than Ref_Mid after 2045, although its impact on aerosol ERF remains limited.

Across all scenarios, the ERF of CO_2_, CH_4_, N_2_O, and contrails follows a nonlinear trend, increasing with altitude, peaking at 34,500 ft, and then declining, as shown in Figures 1E and 1F. Aerosol ERF, in contrast, decreases with altitude until reaching its lowest value at 34,500 ft, after which it begins to increase. Most ERF variations occur at altitudes between 20,500 and 40,500 ft.

Among all scenarios, Pol_Low is the most effective at controlling CO_2_, contrail, and aerosol ERF, followed by H2_Mid, Pol_Mid, and Ref_Mid. For CH_4_, the Pol_Mid scenario shows the strongest control, outperforming H2_Mid and Ref_Mid, further demonstrating the impact of aviation policies on CH_4_ ERF. However, for N_2_O, neither H2_Mid nor Pol_Mid shows significant control, reinforcing the need for stronger mitigation measures.

### Temperature changes in the four scenarios of global aviation

Figures 1G and 1H illustrate the change in temperature at each emission altitude from 500 to 40,500 ft between 2025 and 2050 for the four scenarios.

From Figure 1G, it can be seen that compared with the Ref_Mid scenario, the other two scenarios, except for the Pol_Low scenario, do not control the temperature change significantly and the Pol_Low scenario is the most effective in controlling the temperature, with a temperature control of 8.03E-8°C in 2050, which is a 10% decrease in the temperature increase compared with the 9.12E-8°C of the Ref_Mid scenario. From Figure 1H, it can be seen that the trend of temperature change is generally consistent with the trend of gas concentration and ERF at each emission altitude, the temperature change in the low-altitude environment is relatively smooth and has a small decrease, and with the increase of the emission altitude, the temperature change is also accelerated gradually, in which the strongest effect of the four scenarios on the control of the temperature is still the Pol_Low scenario. After reaching the peak at 34,500 ft, it began to fall back, which shows that the impact of high-altitude emissions on climate change is more significant, further emphasizing the importance of reducing high-altitude emissions.

### Temperature changes across the scenarios

Figures 1G and 1H illustrate the temperature changes at emission altitudes ranging from 500 to 40,500 ft between 2025 and 2050 across the four aviation scenarios. The results indicate that, compared with the Ref_Mid scenario, only the Pol_Low scenario demonstrates a significant impact on temperature control, whereas the other two scenarios show minimal effect. As seen in Figure 1G, the Pol_Low scenario is the most effective in mitigating temperature increases, achieving a temperature reduction of 8.03E-8°C by 2050—a 10% decrease compared with the 9.12E-8°C projected in the Ref_Mid scenario. The H2_Mid and Pol_Mid scenarios, while showing some control, fail to significantly curb temperature rise compared with Ref_Mid. This suggests that without stringent aviation policies or alternative fuels, temperature increases will persist, reinforcing the necessity for proactive climate mitigation strategies. Figure 1H reveals that temperature trends at different altitudes closely align with the variations observed in gas concentrations and ERF. At lower altitudes, temperature changes remain relatively stable with slight fluctuations. However, as the emission altitude increases, the rate of temperature change accelerates, reaching its peak at 34,500 ft. Among the four scenarios, Pol_Low remains the most effective in controlling temperature rise.

Following the peak at 34,500 ft, temperature change begins to decline, indicating that emissions at higher altitudes have a more pronounced impact on climate change. This finding underscores the critical importance of reducing high-altitude emissions as a key strategy for mitigating aviation-related climate effects.

### Cost-benefit analysis of the six scenarios when different clean energies are used

**Figure 2:**
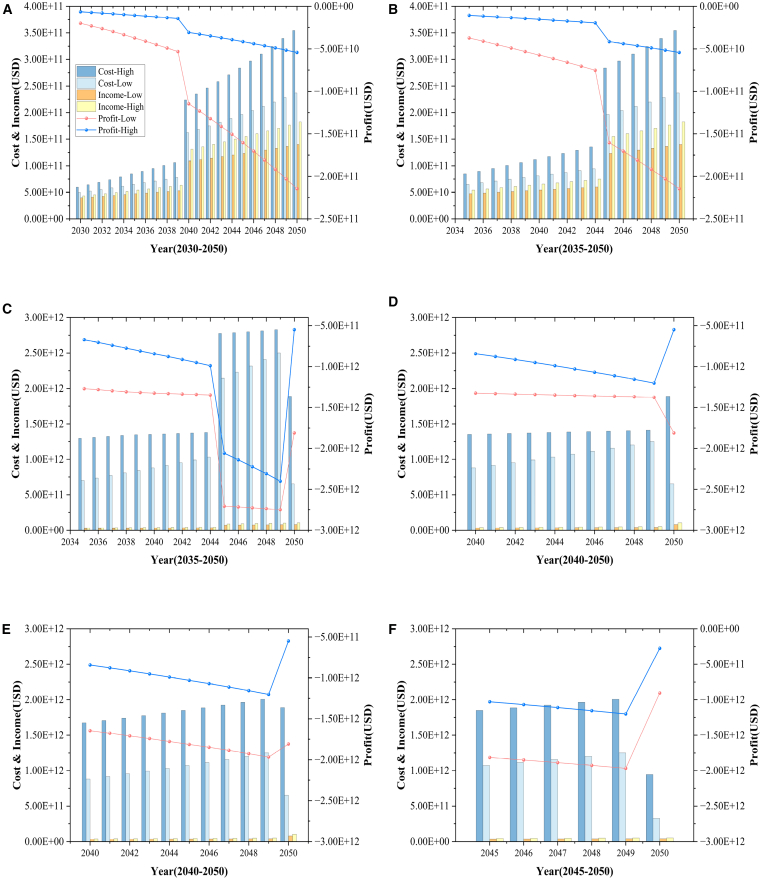
Cost-benefit under six scenarios, 2030–2050 (A) Cost-benefit of using SAF as an alternative energy under Sub-scenario 1. (B) Cost-benefit of using SAF as an alternative energy under Sub-scenario 2. (C) Cost-benefit of using hydrogen turbine engine as an alternative energy under Sub-scenario 3. (D) Cost-benefit of using hydrogen turbine engine as an alternative energy under Sub-scenario 4. (E) Cost-benefit of using hydrogen fuel cell as an alternative energy under Sub-scenario 5. (F) Cost-benefit of using hydrogen fuel cell as an alternative energy under Sub-scenario 6.

The cost-benefit analysis for these six scenarios is illustrated in Figure 2, which evaluates the costs and revenues for each sub-scenario. Figures 2A–2F represent Sub-scenarios 1–6, respectively. The specifics of each program are shown in Table 1. In Sub-scenario 1, which assumes the use of SAF as an alternative energy source starting in 2030, both costs and benefits increase over time. However, the gap between them continues to widen. In 2030, the estimated cost-benefit range spans from −$20.00 billion to −$6.45 billion, showing that costs significantly exceed benefits during the initial phase. This trend persists throughout the following decades, with the gap widening further in 2035 to between −$37.23 billion and −$10.60 billion, and by 2050, it reaches between −$214.40 billion and −$54.16 billion. Sub-scenario 2, which introduces SAF in 2035, follows a similar trend, although its cost and revenue trends remain stable between 2040 and 2044. This stability contrasts with Sub-scenario 1, which sees a sharp rise in both costs and revenues during the same period. This discrepancy can be attributed to the cumulative effects of early adoption, where infrastructure investments and operational adjustments contribute to a rapid escalation in costs and benefits about 10 years after SAF implementation. In Sub-scenario 1, civil aviation revenues would need to reach $223.69 billion in $2,040 and $354.44 billion in 2050 to cover the cost of implementing SAF. In Sub-scenario 2, the required revenues would need to be $111.84 billion in $2,040 and $354.44 billion in 2050.

**Table 1:** The timing and proportion of using hydrogen energy and SAFs in each sub-scenario

Year		2030		2035		2040		2045		2050	
Scenario		SAFs	H	SAFs	H	SAFs	H	SAFs	H	SAFs	H
SAF	Scenario 1	50%	0	50%	0	100%	0	100%	0	100%	0
	Scenario 2	0	0	50%	0	50%	0	100%	0	100%	0
HTE	Scenario 3	0	0	0	20%	0	20%	0	40%	0	40%
	Scenario 4	0	0	0	0	0	20%	0	20%	0	40%
HFC	Scenario 5	0	0	0	0	0	20%	0	20%	0	40%
	Scenario 6	0	0	0	0	0	0	0	20%	0	20%

When considering hydrogen energy, particularly in the form of hydrogen turbine engines (HTEs), the cost implications are even more substantial. First, it is important to highlight the fundamental differences between HTEs and hydrogen fuel cells (HFCs). HTE generates thrust by directly combusting hydrogen in modified gas turbines, whereas HFCs produce electricity through electrochemical reactions to power electric propulsion systems. In Sub-scenario 3, a pattern like SAF emerges, with costs and revenues rising dramatically after a period of operation. However, in this case, costs decline after 5 years of rapid escalation, whereas revenues continue to increase, leading to an improvement in the cost-benefit ratio. For instance, in Sub-scenario 3, the projected cost-benefit in 2035 ranges from −$1,268.16 billion to −$670.53 billion, and in 2040, it further declines to between −$1,322.89 billion and −$842.84 billion. From 2045 onward, both costs and revenues continue to increase, stabilizing over the next 5 years. By 2050, although revenues continue to grow, the cost of retrofitting aircraft decreases significantly, leading to a considerable improvement in cost-benefit, with the projected range shifting to between −$1,808.52 billion and −$548.82 billion. In contrast, Sub-scenario 4 shows a more stable cost-benefit trend between 2040 and 2044. However, from 2045 onward, Sub-scenario 4’s cost-benefit improves more significantly than that of Sub-scenario 3. The projected cost-benefit in 2045 ranges from −$1,352.61 billion to −$1,029.19 billion, and in 2049, it ranges from −$1,374.76 billion to −$1,200.61 billion. By 2050, both Sub-scenarios 3 and 4 converge, with a cost-benefit range of −$1808.52 billion to −$548.82 billion.

For HFCs, Sub-scenario 5 models the cost-benefit of introducing HFCs from 2040 to 2050. In 2040, the initial projection stands at −$1,643.86 billion to −$842.82 billion. Over time, the costs initially decrease, but by 2045, the cost-benefit further declines to between −$1,813.93 billion and −$1,029.17 billion. Although fuel costs decline over the next decade, retrofitting expenses continue to rise, and the rate of fuel cost reduction does not offset the increase in modification costs, resulting in an overall rise in total costs despite increasing revenues. In 2050, whereas fuel costs surge sharply, retrofitting costs decline significantly and the revenue doubles. However, the sharp increase in fuel costs limits the overall improvement in cost-benefit, which is projected to range from −$1,808.47 billion to −$548.76 billion. Sub-scenario 6, which introduces HFCs in 2045, mirrors Sub-scenario 5’s trends from 2045 to 2049 but sees a significant cost reduction in 2050. The reduction in fuel and retrofitting costs, combined with increased revenues, leads to a more substantial improvement in the cost-benefit ratio, projected at −$904.24 billion to −$274.38 billion.

Across all hydrogen scenarios, costs and revenues remain relatively stable in the first 10 years. However, after a decade, costs rise dramatically before either stabilizing or improving by 2050. This trend underscores the early-stage nature of hydrogen technology, where initial infrastructure investments and aircraft modifications drive high costs. As the technology matures, late-stage improvements lead to better cost efficiency. The comparison between Sub-scenarios 3 and 4, as well as between Sub-scenarios 5 and 6, suggests that delaying implementation may help mitigate the initial high costs and reduce long-term conversion expenses. Furthermore, given the goal of achieving NZEs by 2050, hydrogen adoption is expected to accelerate between 2045 and 2049, likely leading to increased transition costs before cost-benefit improvements are realized as the technology matures. In terms of covering costs, civil aviation revenues in Sub-scenarios 3 and 4 would need to reach $1,354.01 billion in 2040 and $1,888.44 billion in 2050. In Sub-scenario 5, revenues would need to be $1,674.99 billion in 2040, with the 2050 requirement the same as in Sub-scenarios 3 and 4. In Sub-scenario 6, revenues would need to reach $944.22 billion in 2050 to cover the cost of using hydrogen energy.

## Discussion

This study conducts a comprehensive analysis using the prediction data from the AIM. It employs the Aviation-FAIR model to simulate changes in atmospheric concentration, ERF, and temperature across emission altitudes ranging from 500 to 40,500 ft from 2025 to 2050. Finally, the AIM is utilized to analyze the economic feasibility of alternative energy technologies, such as SAF, hydrogen turbines, and HFCs, over the same period.

The findings indicate that aviation emissions significantly contribute to climate change. GHG emissions and their climate impact vary considerably across different scenarios. CO_2_, CH_4_, and N_2_O concentrations follow a distinct altitude-dependent pattern: they remain stable at lower altitudes (500–20,500 ft), increase sharply above 20,500 ft, peak at 34,500 ft, and then decline. This pattern suggests that prolonged atmospheric retention at higher altitudes amplifies radiative effects. Under the Ref_Mid scenario, the continuous increase in GHG concentrations and radiative forcing underscores the growing impact of aviation emissions on climate change, highlighting the urgent need for effective policy measures. However, the adoption of specific aviation policies in the Pol_Mid and Pol_Low scenarios, along with hydrogen energy in the H2_Mid scenario, helps curb the growth of CO_2_, CH_4_, and N_2_O concentrations and their radiative effects, demonstrating that policy interventions, demand reduction, and hydrogen adoption can effectively mitigate aviation’s climate impact.

The study also evaluates the cost-benefit analysis of alternative energy sources across six different scenarios, with key time points being 2035, 2040, 2045, and 2050, using the Ref_Mid scenario as the business-as-usual baseline. Across all six scenarios, the cost-benefit remains negative throughout the study period. However, in certain years, such as 2050, scenarios incorporating hydrogen energy exhibit growth. Sub-scenarios 1 and 2, which adopt SAF as an alternative energy source, yield the highest cost-benefit, whereas Sub-scenarios 3 and 4, which use HTEs, show the lowest cost-benefit. Costs and revenues increase at a steady rate in the first 10 years following the introduction of alternative energy sources. After this period, both costs and revenues rise more sharply, with the most significant changes observed in Sub-scenarios 1 and 2. By 2050, a divergence in cost-benefit trends is observed. In Sub-scenarios 1 and 2, costs and revenues continue to rise, leading to a further decline in cost-benefit. However, in the other four scenarios (HTEs and HFCs), costs begin to decline to varying degrees, and for the first time, cost-benefit shows growth. This highlights the economic challenges of reducing carbon emissions with current alternative aviation energy technologies. To cover the costs of SAF and hydrogen energy as alternative energy sources in 2040, civil aviation revenues would need to reach $223.69 billion and $1,674.99 billion, respectively, across all six scenarios. By 2050, the required revenues would need to reach $354.44 billion for SAF and $1,888.44 billion for hydrogen energy to offset the costs across all six scenarios. This indicates that although alternative energy technologies in aviation can help reduce carbon emissions, they still face significant economic challenges.

To support aviation decarbonization, this study recommends a phased policy strategy aligned with the maturity of different technologies. In the near term, governments should promote the adoption of SAF through subsidies and blending mandates, as SAF demonstrates more favorable cost-benefit performance before 2045. For hydrogen-based technologies, targeted investments in infrastructure and research are essential to lower the long-term costs. Introducing carbon pricing mechanisms could further enhance the competitiveness of low-carbon fuels. Moreover, international coordination—such as alignment with CORSIA and Long-Term Aspirational Goal for international aviation frameworks—is vital to ensure policy coherence and advance global emission reduction goals. These actions can help reconcile environmental objectives with economic feasibility, accelerating the aviation sector’s transition to clean energy.

### Conclusion

This study underscores the substantial climate impact of aviation emissions, particularly at higher altitudes where prolonged atmospheric residence time amplifies radiative forcing. Using simulations from the AIM and the Aviation-FAIR model, the analysis reveals a distinct altitude-dependent pattern in GHG concentrations, highlighting the pivotal role of cruise altitudes in climate forcing. Although alternative energy sources such as SAF and hydrogen-based technologies hold promise for emission reduction, their current economic viability remains constrained. Cost-benefit analyses across six scenarios reveal persistent financial challenges—especially for hydrogen technologies, which require considerable revenue to offset implementation costs. In contrast, SAF demonstrates more favorable economic performance over the short to medium term. These findings emphasize the need for a phased policy strategy aligned with the maturity of each technology: promoting SAF adoption in the near term, while advancing hydrogen solutions through sustained investment and infrastructure development. Overall, this research offers a data-driven foundation for formulating targeted policies that reconcile climate objectives with economic feasibility, facilitating a sustainable transition for the aviation sector toward low-carbon energy.

### Limitations of the study

Despite these valuable insights, certain limitations exist in this study. For example, neither were the life cycle emissions of SAF, hydrogen, and electricity fully accounted for nor were the costs associated with retrofitting airports for hydrogen and electric infrastructure included. Future research will address these gaps, incorporating a more comprehensive cost analysis to improve the accuracy and credibility of the findings.

## Resource availability

### Lead contact

Further information and requests should be directed to the lead author, Qiang Cui (cuiqiang@seu.edu.cn).

### Materials availability

This study did not generate new unique materials.

### Data and code availability

• Some basic data are from the results of the AIM model,^34^ some original data and corresponding calculation steps required for this article are shown in the supplemental information, the results of climate change and cost-benefit are available in Tables S1 and S2. See details in the key resources table.
• This study did not generate new code.
• Any additional information required to reanalyze the data reported in this paper is available from the lead contact upon request.

## Acknowledgments

This research is funded by the 10.13039/501100001809National Natural Science Foundation of China (72374042).

## Author contributions

Q.C. designed the study. Q.C., X.-j.S., X.-y.T., Y.Z., Y.-x.Z., and Y.L. performed the analysis and prepared the manuscript. Q.C., X.-j.S., X.-y.T., Y.Z., Y.-x.Z., and Y.L. compiled the original data and participated in writing and revising the manuscript.

## Declaration of interests

The authors declare no competing interests.

## STAR★Methods

### Key resources table

**Table:** 

REAGENT or RESOURCE	SOURCE	IDENTIFIER
**Deposited data**		

Annual emissions data for various scenarios	Dray,^34^ 2025	https://www.atslab.org/wp-content/uploads/2019/12/AIM-2015-Documentation-v9-122019.pdf
Crude oil price	IEA (2025)	https://www.iiea.com/publications
Proportion of four body aircrafts	Oliverwyman (2023)	https://www.oliverwyman.com/content/dam/oliverwyman/v2/publications/2022/feb/MRO-2022-Master-file_v5.pdf

### Method details

#### The method of climate change

Based on the calculation results of the AIM model, this paper obtains the emissions of carbon dioxide, nitrogen oxides, hydrocarbons, carbon monoxide, PM and sulfur dioxide from the global civil aviation industry at various emission altitudes from 500 to 40,500 ft from 2025 to 2050. Then, the Aviation-Fair model is used to calculate the concentration, effective forced radiation and temperature changes at various emission altitudes from 500 to 40,500 ft from 2025 to 2050. The Aviation-Fair model was proposed by Cui and has been widely used in the study of climate change in the civil aviation industry.^25^^,^^37^

Compared with other methods, the Aviation-FAIR model offers several key advantages^37^: (1) it delivers a well-calibrated simulation of temperature and carbon cycle responses within an Earth system model while maintaining high computational efficiency; (2) it monitors the time-integrated fraction of atmospheric carbon, allowing for the assessment of carbon sink efficiency and the subsequent estimation of changes in CO_2_ concentration, radiative forcing, and atmospheric temperature; and (3) it has been enhanced to evaluate concentrations of non-CO_2_ greenhouse gases, further broadening its applicability.

The FAIR model also has some limitations.^38^ First, it primarily focuses on anthropogenic emission sources and simplifies the treatment of natural forcing factors. Additionally, as a globally averaged model, FAIR cannot provide climate predictions at regional or local scales. This limits its applicability in contexts that require high spatial resolution information, such as local climate policy-making and risk assessment. However, since this paper mainly investigates the climate change impacts of aviation emissions and discusses the global aviation industry, these two limitations have little effect on the interpretation of this paper’s results.

#### The cost-benefit analysis method

This study uses the emission results of the AIM model scenario Ref_Mid as the Business as Usual scenario. In addition, this paper also defines six sub-scenarios, two sub-scenarios about SAF, two sub-scenarios about Hydrogen Turbine Engine (HTE), and two sub-scenarios about hydrogen Fuel Cell.

The clean energy options include Sustainable Aviation Fuel (SAF)^39^ and two types of hydrogen-powered engines: Hydrogen Turbine Engine (HTE) and Hydrogen Fuel Cell (HFC) engine.^40^^,^^41^ Airbus aims to achieve 50% SAF mixed commercial use by 2025 and has already tested 100% SAF on mainstream aircraft with the goal of achieving this by 2030.^42^ Besides, Airbus plans to commence commercial use of hydrogen energy in 2035, but the proportion of hydrogen energy may not be significant.^43^^,^^44^

Compared with ordinary kerosene fuel, the CO_2_ and CH_4_ can be reduced by 50%–90%. We assume that CO_2_ and HC will reduce by 70% (the average value) when the SAFs are used. NO_x_ is 0.^42^

Compared with ordinary engines, the application of hydrogen turbine engines will reduce NO_x_ by 50%–80%. At the same time, CO_2_ emission is 0%, HC is 0%.^43^^,^^44^ We assume that NOx will reduce by 65% (the average value) when the hydrogen turbine engines are used.

Compared with ordinary engines, hydrogen fuel cells only produce water, and CO_2_, HC, and NOx emissions are 0%.^43^^,^^44^

Consequently, the following sub-scenarios are established based on the anticipated adoption paths of clean aviation energy. For SAF, two sub-scenarios are assumed: Sub-scenario 1 (SAF-Start in 2030): 2030-50% + 2040-100%; Sub-scenario 2 (SAF-Start in 2035): 2035-50% + 2045-100%. For HTE, two sub-scenarios are assumed: Sub-scenario 3 (HTE-Start in 2035): 2035-20% + 2045-40%; Sub-scenario 4 (HTE-Start in 2040): 2040-20% + 2050-40%. For HFC, two sub-scenarios are assumed: Sub-scenario 5 (HFC-Start in 2040): 2040-20% + 2050-40%; Sub-scenario 6 (HFC-Start in 2045): 2045&HFC20%.

Sub-scenario 1 (SAF-Start in 2030) indicates that 50% of SAF usage will be achieved starting in 2030 and 100% by 2040. This implies that from 2024 to 2029, the aviation activities will rely entirely on conventional jet fuels. From 2030 to 2039, 50% of the energy for aviation activities will come from conventional jet fuels, and the remaining 50% from SAF. From 2040 to 2050, aviation activities will fully depend on SAF.

Sub-scenario 3 (HTE-Start in 2035) indicates that 20% of HTE usage will be achieved starting in 2035 and 40% by 2045. This means that from 2024 to 2034, the aviation activities will rely entirely on conventional jet fuels. From 2035 to 2044, 80% of the energy for aviation activities will come from conventional jet fuels, and the remaining 20% from HTE. From 2045 to 2050, 60% of the energy for aviation activities will come from conventional jet fuels, and the remaining 40% from HTE.

Sub-scenario 5 (HFC-Start in 2040) indicates that 20% of HFC usage will be achieved starting in 2040 and 40% by 2050. This means that from 2024 to 2039, the aviation activities will rely entirely on conventional jet fuels. From 2040 to 2049, 80% of the energy for aviation activities will come from conventional fuels, and the remaining 20% from HFC. In 2050, 60% of the energy for aviation activities will come from conventional jet fuels, and the remaining 40% from HFC.

The cost-benefit calculation method of this article is as follows: the cost of alternative energy transformation mainly consists of two parts: the aircraft transformation cost and the cost difference between alternative energy and JET-A. The benefit is mainly obtained by multiplying the carbon price by the saved carbon dioxide equivalent. That is,

Cost=RenovationCost+AlternativeEnergyCost−JetFuelCost.

$$\text{Renovation}\;\text{Cost}={\text{Unit}\;\text{Renovation}\;\text{Cost}}_{N}*{Number}_{N}+{\text{Unit}\;\text{Renovation}\;\text{Cost}}_{W}*{Number}_{W}+{\text{Unit}\;\text{Renovation}\;\text{Cost}}_{R}*{Number}_{R}+{\text{Unit}\;\text{Renovation}\;\text{Cost}}_{T}*{Number}_{T}$$

${\text{Unit}\;\text{Renovation}\;\text{Cost}}_{N}$ and ${Number}_{N}$ are the unit renovation cost and the number of narrow body aircraft. ${\text{Unit}\;\text{Renovation}\;\text{Cost}}_{W}$ and ${Number}_{W}$ are the unit renovation cost and the number of wide body aircraft. ${\text{Unit}\;\text{Renovation}\;\text{Cost}}_{R}$ and ${Number}_{R}$ are the unit renovation cost and the number of regional aircraft. ${\text{Unit}\;\text{Renovation}\;\text{Cost}}_{T}$ and ${Number}_{T}$ are the unit renovation cost and the number of turboprop.

$$\text{Alternative}\;\text{Energy}\;\text{Cost}={\text{Number}}_{A}*{Price}_{A}\text{.}$$

$$\text{Jet}\;\text{Fuel}\;\text{Cost}={\text{Number}}_{J}*{Price}_{J}\text{.}$$

${\text{Number}}_{A}$ and ${Price}_{A}$ are the number and price of alternative energy, ${\text{Number}}_{J}$ and ${Price}_{J}$ are the number and price of jet fuel.

$$\text{Income}={\text{Saved}\;\text{Number}}_{Carbon}*{Price}_{Carbon}\text{.}$$

${\text{Saved}\;\text{Number}}_{Carbon}$ and ${Price}_{Carbon}$ are the number and price of CO_2_-equivalent. The ${\text{Saved}\;\text{Number}}_{Carbon}$ is the CO_2_-equivalent emission difference between only policy (Pol_Low scenario) and using alternative energy.

CO_2_-equivalent is calculated by the GWP100 method,^45^ which converts methane and nitrous oxide into CO_2_-equivalent.

For basic data on various energy prices, carbon prices, renovation costs, etc., please see the supplemental information for “Altitude-dependent climate impacts and economic feasibility of alternative fuels in aviation from 2025 to 2050”.

### Quantification and statistical analysis

This study does not include statistical analysis or quantification.

## Supplemental information

Document S1. Figures S1, S2, Tables S1–S3, Data S1, and S2

Data S1. Climate change results

Data S2. Cost-benefit results
