# Supplementary material for: Altitude-dependent climate impacts and economic feasibility of alternative fuels in aviation from 2025 to 2050
Source: iScience. 2025 Aug 7;28(9):113323. doi: 10.1016/j.isci.2025.113323 (PMC12496216; doi:10.1016/j.isci.2025.113323)
Supplement: Document S1. Figures S1, S2, Tables S1–S3, Data S1, and S2 [file mmc1.pdf]

iScience, Volume *28*

Supplemental information

Altitude-dependent climate impacts and economic feasibility of alternative fuels in aviation from 2025 to 2050

Qiang Cui, Xu-jie Sun, Xing-yu Tang, Ying Zhou, Yu-xin Zhang, and Ye Li

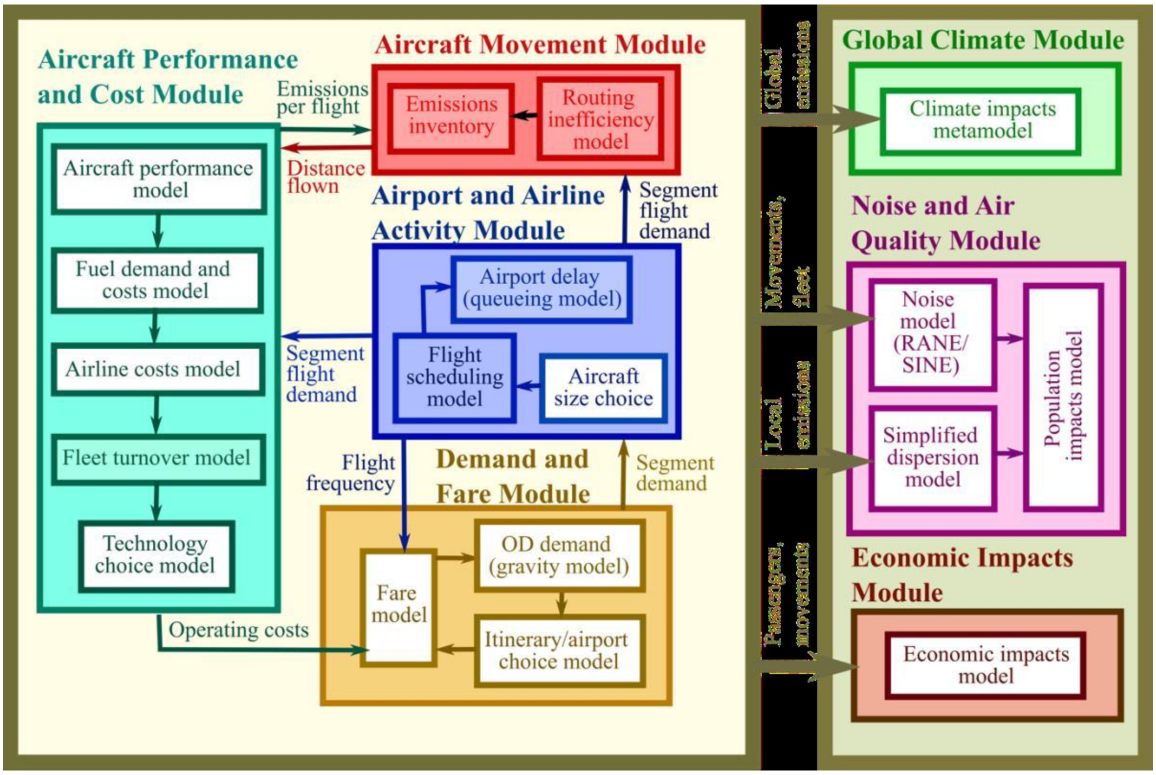

Fig. S1 AIM framework

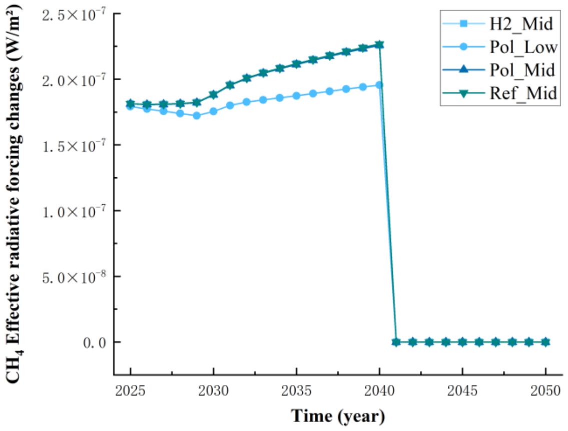

Fig. S2 Effective Radiative Forcing (ERF) changes of CH_4 from 2025 to 2050

Table S1 Price List of Various Types

Year	Jet A-1 ($/barrel)	SAF ($/gallon)	Hydrogen (green hydrogen, $/kg)	Electricity ($/kWh)	Carbon price ($/ton)
1990	39			0.103	
1991	38.9			0.102	
1992	38			0.1	
1993	37			0.089	
1994	38			0.097	
1995	30.2			0.067	

1996	31			0.088	
1997	31			0.077	
1998	37			0.069	
1999	36			0.101	
2000	42			0.104	
2001	46		14.8	0.0833	
2002	49		14.5	0.0912	
2003	54		14.1	0.0921	
2004	60		13.7	0.0943	
2005	99		13.6	0.0966	7.78
2006	124		12.8	0.1112	31.1
2007	142		11.9	0.1175	0.23
2008	162	35	11.5	0.1232	9.67
2009	92	39	10.9	0.1333	8.54
2010	76	15	11.4	0.1557	8.12
2011	96	13.6	10.6	0.1245	7.86
2012	110	14.2	10.2	0.1356	7.5
2013	118	11.9	9.9	0.1422	6.06
2014	70	12.8	9.55	0.1156	6.76
2015	66	13.7	8.39	0.1067	7.69
2016	52	9.87	8.15	0.1324	4.88
2017	76	8.12	7.34	0.1261	6.24
2018	84	6.45	7.06	0.1132	16.37
2019	89	6.76	6.14	0.1167	24.51
2020	26	4.67	5.87	0.1078	18.54
2021	79	4.35	5.74	0.1667	49.78
2022	96	4.26	4.99	0.2045	86.53
2023	115	3.97	4.68	0.2134	95.2086

Table S2 Forecast values of various prices from 2024 to 2050

Year	Jet A-1 ($/barrel)	Jet A-1 ($/barrel)	SAF ($/gallon)	Hydrogen (green hydrogen, $/kg)		Carbon price ($/ton)	
	Low	High		Low	High	Low	High
2024	97.231	98.781	3.99	4.68	10.00	95.94	97.24
2025	94.964	98.066	4.01	4.59	9.77	96.67	99.27
2026	92.698	97.350	4.03	4.50	9.54	97.41	101.30
2027	90.431	96.634	4.06	4.41	9.31	98.14	103.33
2028	88.165	95.919	4.08	4.31	9.08	98.87	105.36
2029	85.898	95.203	4.10	4.22	8.85	99.61	107.38
2030	83.632	94.487	4.12	4.13	8.62	100.34	109.41
2031	81.365	93.771	4.14	4.04	8.38	101.07	111.44
2032	79.099	93.056	4.16	3.95	8.15	101.81	113.47
2033	76.832	92.340	4.18	3.86	7.92	102.54	115.50

2034	74.566	91.624	4.21	3.76	7.69	103.27	117.53
2035	72.299	90.908	4.23	3.67	7.46	104.00	119.56
2036	70.033	90.193	4.25	3.58	7.23	104.74	121.59
2037	67.766	89.477	4.27	3.49	7.00	105.47	123.62
2038	65.500	88.761	4.29	3.40	6.77	106.20	125.65
2039	63.233	88.045	4.31	3.31	6.54	106.94	127.68
2040	60.967	87.330	4.34	3.22	6.31	107.67	129.71
2041	58.700	86.614	4.36	3.12	6.08	108.40	131.74
2042	56.434	85.898	4.38	3.03	5.85	109.14	133.77
2043	54.167	85.183	4.40	2.94	5.62	109.87	135.79
2044	51.901	84.467	4.42	2.85	5.38	110.60	137.82
2045	49.634	83.751	4.44	2.76	5.15	111.33	139.85
2046	47.368	83.035	4.46	2.67	4.92	112.07	141.88
2047	45.101	82.320	4.49	2.57	4.69	112.80	143.91
2048	42.835	81.604	4.51	2.48	4.46	113.53	145.94
2049	40.568	80.888	4.53	2.39	4.23	114.27	147.97
2050	38.302	80.172	4.55	2.30	4.00	115.00	150.00

Table S3 Average hydrogen renovation cost for the aircraft

Year	wide-body		narrow-body		Regional aircraft		Turboprop	
	Low	High	Low	High	Low	High	Low	High
2024	100,000,000	300,000,000	50,000,000	150,000,000	20,000,000	80,000,000	10,000,000	50,000,000
2050	50,000,000	150,000,000	25,000,000	75,000,000	10,000,000	40,000,000	5,000,000	25,000,000

Data S1

Introduction to the AIM model and its forecasts

This study primarily draws on the results of AIM v11, which incorporates multiple aviation-related modules. Using 2015 data as a baseline, it simulates outcomes for four scenarios[1]. The framework of the model is illustrated in Fig. S1.

It defines four key scenarios:

① Ref_Mid: Assumes no major aviation policies or adoption of alternative fuels.

② Pol_Mid: Simulates the impact of specific aviation policies, including CORSIA (expected to take effect in early 2023), the EU and UK ETS, and medium demand growth.

③ Pol_Low: Maintains the same policies as Pol_Mid but assumes a lower demand growth rate.

④ H2_Mid: Considers the adoption of hydrogen energy under medium demand growth and fluctuating oil prices.

The Aviation-FAIR method

According to the FAIR method, CO_2 would be partitioned into four boxes: geological processes (τ_0), the deep ocean (τ_1), the biosphere (τ_2), and the ocean mixed layer (τ_3). The partition fractions are α_i and $\sum_{i=0}^{3} \alpha_i = 1$. The concentration of CO_2 is

$$C_{CO_2} = 278 + \sum_{i=0}^{3} \frac{R_i}{M_a} \frac{\omega_{CO_2}}{\omega}$$

$M_a = 5.1352 * 10^{18}$ kg is the dry mass of the atmosphere, $\omega_{CO_2} = 44.01$ is the molecular weight of CO_2, and $\omega = 28.966$ is the molecular weight of dry air.

In equation (1), the formula of R_i is

$$\frac{dR_i}{dt} = \alpha_i E_{CO_2} - \frac{R_i}{\partial \tau_i}, \quad i = 0,1,2,3$$

E_{CO_2} is the CO_2 emission.

The variable ∂ is gotten by

$$\sum_{i=0}^{3} \partial\, \alpha_i \tau_i \left[1 - \exp(\frac{-100}{\partial \tau_i})\right] = r_0 + r_C\left[\sum_t E_{CO_2,t} - C_{CO_2} + 278\right] + r_T \Delta T$$

r_c =0.019 yt GtC^{-1}, r_T =4.165 yr K^{-1}, and $r_0 = 35$. ΔT is the temperature change.

The formulas (1) - (3) form a cycle, and this study uses one hundred thousand Monte Carlo simulations to solve them.

As part of CH_4 and NO_x, for CH_4 and N_2O, their concentrations are

$$C_t = C_{t-1} + \frac{1}{2}(\delta C_{t-1} + \delta C_t) - C_{t-1}\left(1 - \exp\left(-\frac{1}{\tau}\right)\right)$$

τ the atmospheric lifetime. For CH_4, $\tau = 9.3$; For N_2O, $\tau = 121$. This study sets $C_{2022} = 0$, and the results show the concentration change.

δC_t can be gotten by

$$\delta C_t = \frac{E_t}{M_a} \frac{\omega}{\omega_f}.$$

ω_f is the molecular mass. For CH_4, $\omega_f = 16.04$; For N_2O, $\omega_f = 44.01$. E_t is CH_4 or N_2O emission of year t.

The Effective Radiative Forcing (ERF) of CO_2, N_2O and CH_4 are

$$F_{CO_2} = \left[(-2.4 \times 10^{-7})(C_{CO_2} - C_{CO_2pi})^2 + (7.2 \times 10^{-4})\left|C_{CO_2} - C_{CO_2pi}\right| - (1.05 \times 10^{-4})(C_{N2O} + C_{N_2Opi}) + 5.36\right] \times \log(\frac{C_{CO_2}}{C_{CO_2pi}}).$$

$$F_{N_2O} = \left[(-4.0 \times 10^{-6})(C_{CO_2} + C_{CO_2pi})^2 + (2.1 \times 10^{-6})(C_{N_2O} + C_{N_2Opi}) - (2.45 \times 10^{-6})(C_{CH_4} + C_{CH_4pi}) + 0.117\right] \times (\sqrt{C_{N_2O}} - \sqrt{C_{N_2Opi}}).$$

$$F_{CH_4} = \left[(-6.5 \times 10^{-7})(C_{CH_4} + C_{CH_4pi})^2 - (4.1 \times 10^{-6})(C_{N_2O} + C_{N_2Opi}) + 0.043\right] \times (\sqrt{C_{CH_4}} - \sqrt{C_{CH_4pi}}).$$

C_{CO_2pi}, C_{CH_4pi}, C_{N_2Opi} are the benchmark concentration of CO_2, N_2O and CH_4. Because focusing on the ERF change, so this study sets them as the concentration.

The ERF of Contrails is

$$F_{con} = 0.0152 \times E_{NOx}/M_a.$$

The ERF of Aerosols is

$$F_{aer} = \left(0.08 \times E_{PM_{2.5}} - 0.34 \times E_{SO_2} - 0.044 \times E_{NOx}\right)/M_a.$$

$E_{PM_{2.5}}$, E_{SO_2}, and E_{NOx} are the emissions of PM2.5, SO_2 and NO_x.

The temperature change is

$$\Delta T_t = \Delta T_{t,1} \exp\left(\frac{1}{d_1}\right) + (1 - \exp(\frac{1}{d_1})) \times q_1 \times (F_{CO_2} + F_{N_2O} + F_{CH_4} + F_{con} + F_{aer}) + \Delta T_{t,2} \exp\left(\frac{1}{d_2}\right) + (1 - \exp(\frac{1}{d_2})) \times q_2 \times (F_{CO_2} + F_{N_2O} + F_{CH_4} + F_{con} + F_{aer}).$$

$d_1 = 239$ and $d_2 = 4.1$ indicate the responses to forcing from the upper ocean and the deep ocean. q_1 and q_2 are the coefficients, and in this study, q_1 =3.8729E-04, $q_2 = -3.836$E-04.

Data S2

The basis data for cost-benefit analysis

The first part is forecasting various price trends. This study focuses on forecasting key prices, including those of aviation kerosene, hydrogen energy, sustainable aviation fuel (SAF), and electricity. Drawing on existing literature[2], historical prices for these energy sources from 1990 to 2023 were compiled. Since the hydrogen energy used in aviation primarily consists of green hydrogen, this study specifically analyzes the price of green hydrogen. Electricity prices are averaged across major regions, including China, the United States, and Europe.

In addition to energy prices, carbon trading prices are also a critical factor. These are based on the pricing trends within the EU carbon trading system. Detailed price data can be found in Table S1.

This paper predicts various prices from 2024 to 2050. First, for JET-A, this paper uses the formula of the AIM model to calculate. That is,

$$P_{jet}=0.2062+0.793*(P_{oil}/\text{Base } P_{oil}).$$

P_{jet} is the price of JET-A, P_{oil} is the price of crude oil, and Base P_{oil} is the price of crude oil in 2015. Therefore, to predict the price of JET-A, we only need to get the predicted value of crude oil price. This paper collects the forecast results of crude oil price by the US Department of Energy and substitutes them into the above formula to get the price range of JET-A from 2024 to 2050, as shown in Table S2.

For the price of sustainable aviation fuel (SAF), this study directly adopts the forecast results from the AIM model, as detailed in Table S1. The price range for green hydrogen is derived from existing literature, providing a comprehensive reference for future projections (see Table S2).

For electricity prices, this study follows the AIM model's assumption, maintaining a constant price of USD 0.04/kWh. The forecast for carbon trading prices is also based on existing research, with annual data projections provided in Table S2.

Then, we will predict the aircraft renovation costs. Globally, aircraft in the civil aviation industry are generally classified into four categories: wide-body aircraft, narrow-body aircraft, regional aircraft, and turboprop aircraft. Among the various energy sources, sustainable aviation fuel (SAF) can be used without requiring aircraft renovations. In contrast, hydrogen and electric energy necessitate aircraft renovations, with significant cost differences across the different aircraft types.

Drawing on existing studies of renovation costs for 2024 and 2050 [3,4], this paper estimates the cost ranges for modifying various aircraft types to accommodate different energy sources. The

detailed estimates are provided in Table S3.

If the aircraft uses SAF, there is no need to modify the aircraft. However, if hydrogen energy is used, modification is required. This section introduces the specific calculation method. According to the data in 2023, wide-body aircraft account for about 28% of global aviation emissions, narrow-body aircraft account for about 62%, regional aircraft account for about 7%, and turboprop aircraft account for about 3% [5]. This article assumes that this proportion will remain unchanged from 2024 to 2050. According to the data in 2023, the annual carbon emissions of a single wide-body aircraft are 52,810.18 tons, narrow-body aircraft are 36,204.88 tons, regional aircraft are 19,746.87 tons, and turboprop aircraft are 12,341.4 tons [6].

This article assumes that this data remains unchanged, and calculates the number of wide-body aircraft, narrow-body aircraft, regional aircraft and turboprop aircraft that need to be modified from 2024 to 2050 based on the difference in carbon emissions between various scenarios and the baseline (BAU) scenario.

Energy conversion: JET-A and SAF have the same energy density, so they are equivalent; the energy of one ton of green hydrogen is approximately equivalent to the energy of 2.79 tons of Jet-A fuel[7]; the energy of one ton of Jet-A fuel is approximately equivalent to 11,944 kilowatt-hours (kWh) of electricity[8].

According to the GWP method, the CO_2-equivalent emissions are

$$E_{CO_2} = CO_2 + 25 * CH_4 + 298 * N_2O.$$

Based on existing literatures, this study assumes that the proportion of CH_4 in aviation HC is 0.4, and the proportion of N_2O in aviation NOx is 0.29[9]. The ratio of CO_2-equivalent emissions to CO_2 emissions in GWP100 is 1.7.

The reduced CO_2-equivalent emissions are

$$RE_{CO_2} = \text{Max}\ (E_{CO_2}) - E_{CO_2}.$$

Supplementary Figures

Fig. S2 shows Effective Radiative Forcing (ERF) changes of CH_4 under four scenarios between 2025 and 2050.

References

1. Dray L. AIM2015: Documentation. https://www.atslab.org/wp-content/uploads/2019/12/AIM-2015-Documentation-v9-122019.pdf (2025)

2. IEA. Energy price. https://www.iiea.com/publications (2025).

3. Sky C. Hydrogen-powered aviation: a fact-based study of hydrogen technology, economics, and climate impact by 2050. (2020).

4. Adu-Gyamfi B A, Good C. Electric aviation: A review of concepts and enabling technologies. Transportation Engineering, 9, 100134 (2022).

5. Cooper T, Smiley J, Porter C, et al. Global fleet & MRO market forecast commentary. Olyver Wyman (2018)

6. IATA. IATA annual review 2023. https://www.iata.org/contentassets/c81222d96c9a4e0bb4ff6ced0126f0bb/annual-review-2023.pdf (2025)

7. Yusaf T, Mahamude A S F, Kadirgama K, et al. Sustainable hydrogen energy in aviation–A narrative review. international journal of hydrogen energy, 52, 1026-1045 (2024).

8. Trofimov I L, Iavniuk A A, Radomska M M. Research of the JET A-1 aircraft fuel electrification. International Journal of Sustainable Aviation, 4(3-4), 273-289 (2018).

9. Cui Q, Lei Y. Pathways analysis to reducing aircraft emissions for China-Foreign routes. npj Climate Action, 2(1), 15 (2023).
